# Feline immunodeficiency virus (FIV) *env* recombinants are common in natural infections

**DOI:** 10.1186/s12977-014-0080-1

**Published:** 2014-09-17

**Authors:** Paweł M Bęczkowski, Joseph Hughes, Roman Biek, Annette Litster, Brian J Willett, Margaret J Hosie

**Affiliations:** Centre for Virus Research, University of Glasgow, Glasgow, United Kingdom; Small Animal Hospital, University of Glasgow, Glasgow, United Kingdom; Boyd Orr Centre for Population and Ecosystem Health & Institute of Biodiversity, Animal Health & Comparative Medicine, University of Glasgow, Glasgow, United Kingdom; Department of Veterinary Clinical Sciences, Purdue University, West Lafayette, IN 47907 USA

**Keywords:** FIV, Feline immunodeficiency virus, Recombination, Leader, Quasispecies, Phylogenetic classification, Natural infection

## Abstract

**Background:**

Recombination is a common feature of retroviral biology and one of the most important factors responsible for generating viral diversity at both the intra-host and the population levels. However, relatively little is known about rates and molecular processes of recombination for retroviruses other than HIV, including important model viruses such as feline immunodeficiency virus (FIV).

**Results:**

We investigated recombination in complete FIV *env* gene sequences (n = 355) isolated from 43 naturally infected cats. We demonstrated that recombination is abundant in natural FIV infection, with over 41% of the cats being infected with viruses containing recombinant *env* genes. In addition, we identified shared recombination breakpoints; the most significant hotspot occurred between the leader/signal fragment and the remainder of *env*.

**Conclusions:**

Our results have identified the leader/signal fragment of *env* as an important site for recombination and highlight potential limitations of the current phylogenetic classification of FIV based on partial *env* sequences. Furthermore, the presence of abundant recombinant FIV in the USA poses a significant challenge for commercial diagnostic tests and should inform the development of the next generation of FIV vaccines.

**Electronic supplementary material:**

The online version of this article (doi:10.1186/s12977-014-0080-1) contains supplementary material, which is available to authorized users.

## Background

Recombination, together with point mutations introduced by the error prone reverse transcriptase (RT) [1] and the activity of host restriction factors [2], is regarded as the most important mechanism for generating genetic diversity among retroviruses [3,4]. Two features of the retroviral life cycle facilitate recombination: 1) the presence of two RNA genomes within each viral particle and 2) the tendency of RT to switch between those RNA molecules during provirus synthesis [5,6]. This can result in the synthesis of recombinant DNA of mixed ancestry, originating from both RNA molecules [7]. In HIV-1 infection, recombination occurs between homologous viral variants of similar fitness at an exceptionally high rate [8,9]. However, its significance in generating novel variants increases dramatically when a cell becomes infected with two or more genetically distinct virions [10]. Recombination can occur between virions of the same or different subtypes, resulting in the generation of intra- or inter-subtype recombinants, respectively [10]. Recombination can have profound implications on the fitness of the generated viral quasispecies and subsequently on the pathogenesis and clinical outcome of infection [11]. Intra- and inter-subtype recombinants have been identified in HIV infection and unique recombinant forms (URF) with limited transmission can be distinguished from commonly circulating recombinant forms (CRF), where the term CRF applies to recombinant viruses sharing identical mosaic structures that are detected in multiple distinct epidemiological areas [12]. CRFs play an important role in the HIV pandemic, accounting for over 20% of infections in some countries [13]. In contrast to HIV, much less is known about natural recombination in other retroviruses, including important models such as feline immunodeficiency virus (FIV). Although FIV recombinants have been identified [14-17], the prevalence of recombinant *env* sequences remains poorly quantified. This applies particularly to the open reading frame of the *env* gene which, in contrast to primate retroviruses, contains an unusually long leader/signal region [18].

FIV is currently assigned to five distinct subtypes, denoted A, B, C, D and E, based on the diversity of the V3-V5 region of the *env* gene [19]. Subtype A is prevalent in the west coast of the USA and Australia and is the only subtype found in the UK, while subtype B is most prevalent on the east coast of the USA and central and southern Europe [20-24]. Subtype C has been identified in California [20], Canada [14,16] and Taiwan [25] and subtype D in Japan [19,26], while the putative subtype E was reported in Argentina [27]. Hence, despite extensive movement of humans and their cats and subtypes A, B and C being found on multiple continents [14], geographical clustering of FIV (based on the current classification) is still evident. However, the vast majority of published *env* sequences represent a V3-V5 region of approximately 700 bp*,* which is too short to enable the reliable detection of recombination.

Given that most previous studies focused upon relatively small gene fragments, the occurrence and role of recombination in natural FIV infection could well have been underestimated. The aim of this study was to address this issue by studying the molecular evolution and recombination of FIV in two cohorts of cats in the USA (Memphis, n = 27 and Chicago, n = 16) naturally infected with FIV. By examining FIV *env* sequences from 43 domestic cats naturally infected with FIV, we aimed to determine a) the prevalence of recombinant *env* sequences; b) the subtype composition amongst field isolates; and c) the site(s) of common recombination break-points.

## Results

### Phylogenetic inference

A Maximum Likelihood (ML) tree was constructed using the entire data set [see Additional file 1] and examined carefully for evidence of non-monophyletic clustering of multiple sequences isolated from each cat. Intra-host sequences isolated from the majority of cats (n = 40, 93%) clustered together, forming monophyletic groups. After excluding data from the three cats with inconsistent phylogenetic assignment, analysis of the tree revealed that sequences from 24 cats clustered together with previously published clade B reference sequences, while sequences from 16 cats were assigned to clade A. The number of *env* genes classified as clade A (7/14, 50%) and B (7/14, 50%) were equally distributed in the Chicago cats, while in the Memphis cohort, a larger proportion of *env* genes belonged to clade B (17/26, 65%). Sequences (comprising 7% of the total) from three cats (M5, P8 and P21) isolated from different time points showed inconsistent phylogenetic assignments, suggesting that they might have been infected with additional viruses, perhaps transmitted from cats not sampled for this study, or that sample sizes at later time points were insufficient to detect the earlier virus.

**Figure 1:**
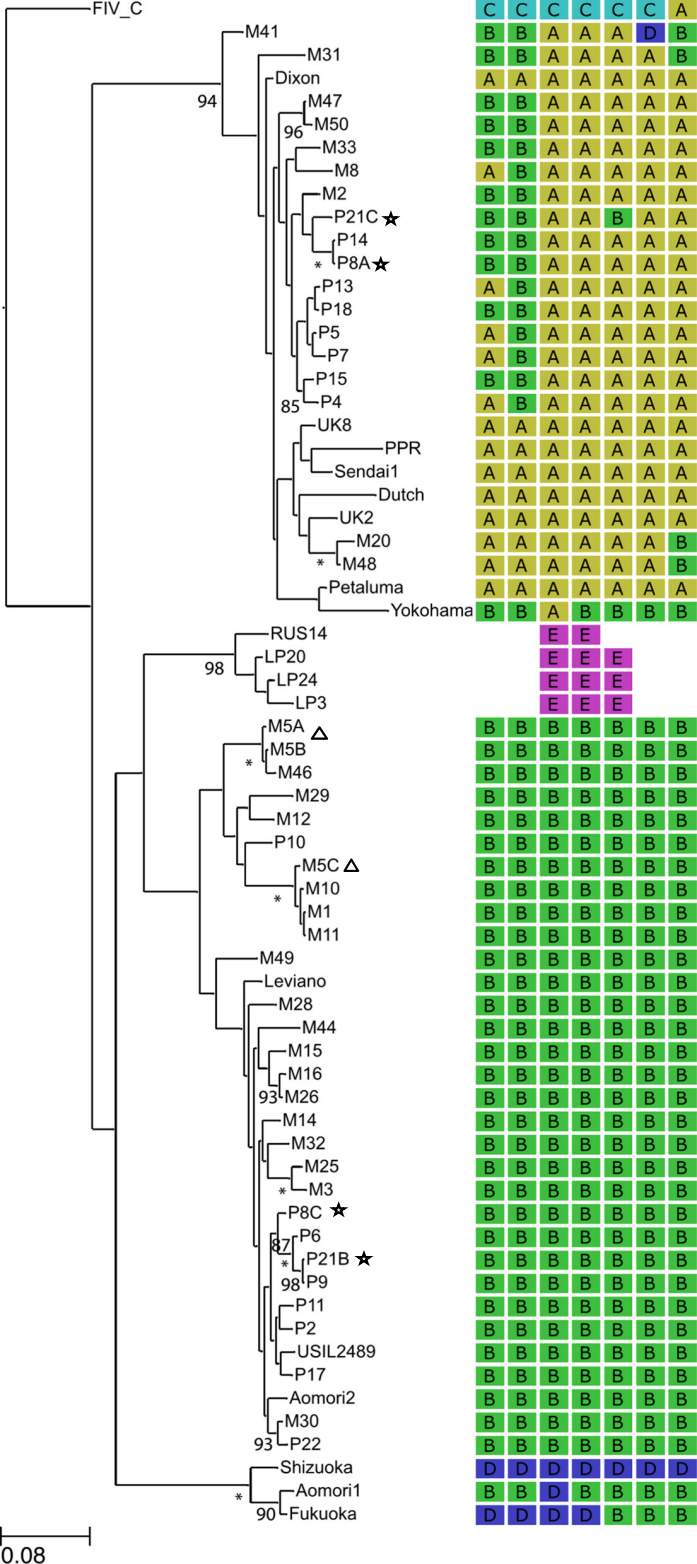
**Maximum likelihood (ML) tree based on the 708 bp fragment (span 3 as identified by GARD) of the** ***env*** **sequences and their recombination history as inferred by GARD.** The data include 47 entire *env* nucleotide sequences (representative of a total of 355 sequences from Chicago and Memphis), 15 full length *env* sequences derived from GenBank: Aomori 1 [D37816], Aomori 2 [D37817.1], FIV C [AF474246.1], Dixon [L00608.1], Dutch [X60725], Fukuoka [D37815.1], Sendai 1 [D37813.1], Shizuoka [D37811.1], UK2 [X69494.1], UK8 [X69496.1], USIL2489 [U11820.1], Yokohama [D37812.1], Petaluma [M25381.1], PPR [M36968.1], Leviano [FJ374696.1], three V3-V5 region sequences representing Clade E: LP3 [D84496], LP20 [D84498], LP24 [D84500] and one shorter 504 bp in length RUS14 [EF447297] sequence. Taxa with inconsistent clade assignment are represented with an asterisk (P8, P21). Non-monophyletic taxa from cat M5 are marked with a triangle. The tree is based on an HKY model, rooted on FIV C sequence and is drawn to scale, with branch lengths measured in substitutions per site. Only bootstrap values above 80 are shown. * represents bootstrap values of 100.

The ML tree from the entire dataset was used to select a reduced data set for recombination analysis comprising: a) one randomly selected sequence from each cat where the intra-host sequences formed a monophyletic cluster, and b) all sequences from non-monophyletic groups. These sequences were combined with 19 reference sequences and used to construct a further ML tree which was rooted on the reference clade C *env* sequence [Figure 1].

### Recombination

GARD analysis of the data set (n = 68) identified 22 recombinants providing evidence for four breakpoints with significant topological incongruence (p = 0.01). A subsequent GARD analysis was run on the data set comprising only reference sequences and previously identified recombinant sequences (n = 22). This alignment was spliced by GARD at six breakpoints into seven spans: 1) 1-354; 2) 355-564; 3) 565-1272; 4) 1273-1608; 5) 1609-1868; 6) 1869-2255 and 7) 2256-2604 [Figure 1]. For each span, a separate ML tree was created. Examination of GARD-determined trees [see Additional file 2: Figure S1] revealed that sequences from 18/26 (69%) of the Memphis cats showed consistent clade assignment and represented non-recombinant clade B *envs*, whereas recombinant sequences were identified in the remaining 8/26 (31%) cats. In the Chicago group, sequences from 7/14 cats (50%) were non-recombinant and consistently clustered with the reference clade B *env* sequences, while the remaining 7/14 sequences (50%) were identified as A/B recombinants.

The jpHMM analysis generally supported all of the previous recombination breakpoints identified in GARD, but also suggested the existence of seven additional breakpoints [see Additional file 3: Table S1]. In order to focus on the most strongly supported recombination events, we examined in detail the breakpoints identified by the two methods.

Recombinant sequences had different mosaic compositions and were classified in three groups: 1) sequences from cats M2, M33, M47, M50, P14, P15 and P18 in which the first two spans (1-354, 355-564) belonged to clade B, while the remaining five spans consistently clustered as clade A; 2) sequences from cats M8, P4, P5, P7 and P13 where only the second span (355-564) was assigned to clade B while the remaining spans clustered with clade A; and 3) sequences from cats M20 and M48 in which only the last span (2256-2604) was assigned to clade B, while all other fragments were assigned to clade A. The remaining two recombinant sequences (from cats M31 and M41) did not have a recombination pattern in common with any of the other sequences in the data set [Table 1]. The locations of the recombination hotspots between the leader and the stem of the V1/V2 region (approximately position 562-576) were common among recombinants from both cohorts [Table 1]. A similarity plot representative of the Memphis and Chicago recombinants in relation to reference A and B *env* sequences is shown in [see Additional file 4: Figure S2].

**Table 1:** **Recombination testing of sequences from Memphis and Chicago**

	**GARD determined breakpoints**							**RDP determined recombinants**		
***env***	**S1**	**S2**	**S3**	**S4**	**S5**	**S6**	**S7**	**CLADE**	**MINOR PARENT**	**MAJOR PARENT**
	**1-354**	**355-564**	**565-1272**	**1273-1608**	**1609-1868**	**1869-2255**	**2256-2604**			
M1	B	B	B	B	B	B	B	B	NR	NR
M2	B	B	A	A	A	A	A	A/B	P10	DIXON
M3	B	B	B	B	B	B	B	B	NR	NR
M5A†	B	B	B	B	B	B	B	B	NR	NR
M5C†	B	B	B	B	B	B	B	B	NR	NR
M8	A	B	A	A	A	A	A	A/B	P10	DIXON
M10	B	B	B	B	B	B	B	B	NR	NR
M11	B	B	B	B	B	B	B	B	NR	NR
M12	B	B	B	B	B	B	B	B	NR	NR
M14	B	B	B	B	B	B	B	B	NR	NR
M15	B	B	B	B	B	B	B	B	NR	NR
M16	B	B	B	B	B	B	B	B	NR	NR
M20	A	A	A	A	A	A	B	A/B	P2	DIXON
M25	B	B	B	B	B	B	B	B	NR	NR
M26	B	B	B	B	B	B	B	B	NR	NR
M28	B	B	B	B	B	B	B	B	NR	NR
M29	B	B	B	B	B	B	B	B	NR	NR
M30	B	B	B	B	B	B	B	B	NR	NR
M31	B	B	A	A	A	A	B	A/B	P10	DIXON
M32	B	B	B	B	B	B	B	B	NR	NR
M33	B	B	A	A	A	A	A	A/B	P10	DIXON
M41	B	B	A	A	A	D	B	A/B/D	P10	DIXON
M44	B	B	B	B	B	B	B	B	NR	NR
M46	B	B	B	B	B	B	B	B	NR	NR
M47	B	B	A	A	A	A	A	A/B	P10	DIXON
M48	A	A	A	A	A	A	B	A/B	P2	DIXON
M49	B	B	B	B	B	B	B	B	NR	NR
M50	B	B	A	A	A	A	A	A/B	P10	DIXON
P2	B	B	B	B	B	B	B	B	NR	NR
P4	A	B	A	A	A	A	A	A/B	P10	DIXON
P5	A	B	A	A	A	A	A	A/B	P10	DIXON
P6	B	B	B	B	B	B	B	B	NR	NR
P7	A	B	A	A	A	A	A	A/B	P10	DIXON
P8A†	B	B	A	A	A	A	A	A/B	P10	DIXON
P8C†	B	B	B	B	B	B	B	B	NR	NR
P9	B	B	B	B	B	B	B	B	NR	NR
P10	B	B	B	B	B	B	B	B	NR	NR
P11	B	B	B	B	B	B	B	B	NR	NR
P13	A	B	A	A	A	A	A	A/B	P10	DIXON
P14	B	B	A	A	A	A	A	A/B	P10	DIXON
P15	B	B	A	A	A	A	A	A/B	P10	DIXON
P17	B	B	B	B	B	B	B	B	NR	NR
P18	B	B	A	A	A	A	A	A/B	P10	DIXON
P21B†	B	B	B	B	B	B	B	B	NR	NR
P21C†	B	B	A	A	B	A	A	A/B	P10	DIXON
P22	B	B	B	B	B	B	B	B	NR	NR

Spans (S1-S7) are assigned as clade A, B and D as inferred by GARD analysis. The final clade assignment following recombination testing is shown in the column “CLADE”. Non recombinant sequences are denoted NR. RDP determined major and minor parents for mosaic recombinant sequences are shown in the final two columns. † sequences from the same cat but different time points with previously identified non-monophyletic clustering.

RDP software was used to examine putative parents contributing to the mosaic structure of recombinant sequences. RDP consistently identified the clade A, FIV Dixon strain [GenBank:L00608.1] as a putative major parent and determined Chicago strains P10 and P2 as putative minor parents for M2, M31, M33, M47, M50, P4, P5, P7, P13, P14, P15, P18 and M20, M48, respectively [Table 1].

Recombination analysis of *env* sequences from three cats carrying non-monophyletic groups of viruses revealed that sequences from cat P8 at time point C were non-recombinant clade B, while time point A sequences were classified as A/B recombinants. It is intriguing that all time point C sequences in this cat contained premature stop codons [see Additional file 5: Table S2]. Taken together with the FIV load data (time point A, 395 genomes/mL; time point C, 1061881 genomes/mL [see Additional file 6: Table S3]), it is possible that cat P8 became infected with freshly acquired clade B *env* virus that outcompeted the recombinant strain. In cat P21, sequences at time point B appeared to be entirely clade B, while those at time point C showed an incongruent assignment and were identified as clade A/B mosaics [Table 1]. A similar scenario to P8 is possible; in this case, recombinant virus (time point C FIV load, 1077 genomes/mL) could have outcompeted the non-recombinant strain from time point B (FIV load, 45 genomes/mL) [see Additional file 6: Table S3]. In contrast, all sequences from cat M5 were non-recombinant [Figure 1]. Examination of their phylogenetic assignment, considering the shared housing conditions, suggests that one-way transmission might have occurred during the study. Similar to P8 and P21, the original strain could have been outcompeted by newly acquired virus by time point C (sequential FIV load data were not available for this cat).

There was a notable difference in terms of the abundance of clade A recombinants compared to very few recombinant sequences clustering with clade B sequences [Figure 1]. Based on the ML phylogeny and the recombination analyses of sequences from both cohorts, we propose that the following groups of mosaic viruses represent putative CRFs of FIV: 1) M47, M50 (bootstrap support 96%), 2) M20, M48 (bootstrap support 100%), 3) P14, P8A (bootstrap support 100%). Given that cats in each of those three groups could have previously been involved in territorial fights, it is likely that the proposed CRFs of FIV were transmitted in the field. Although additional clusters of recombinant forms exist, the bootstrap supports were too low to define them confidently.

## Discussion

The phylogenetic relationship of the sequences from Chicago and Memphis cohorts was in agreement with previously published data demonstrating the presence of clade A and clade B viruses in the USA [20]. However, most previous analyses did not account for potential recombination. Our analyses show that *env* genes with shared recombination breakpoints and with inconsistent clade assignment circulated widely in the Memphis and Chicago cohorts, with over 41% of tested cats being infected with a recombinant form.

Recombination is an important event in retroviral evolution, which, in cases of super-infection, can lead to the emergence of novel viral variants. Although there was no evidence of more severe clinical manifestations in cats infected with recombinant strains [28], newly created recombinants could potentially exhibit novel pathogenicity compared to the parental strains, for example manifesting more severe clinical outcomes or being transmitted more easily within and among hosts [4]. Cats with outdoor access, especially in areas where the prevalence of FIV is relatively high, are likely to acquire viruses during multiple transmission events. This phenomenon has been documented in experimental FIV infection [29], with one study describing recombination following super-infection [30]. Recombinant sequences in the present study were consistently identified in subsequent follow up samples, suggesting that such genotypes did not arise as a result of either PCR errors or contamination and that the recombinant viruses incurred no fitness costs preventing them from replicating and persisting over several months. Indeed, the high frequency of clade A/B recombinants in our study might indicate that these represent widely distributed viruses with a significant fitness advantage. A similar pattern of recombination, with a shared break point within *gag,* was reported in a Canadian study, suggesting the existence of enzootic recombinant forms circulating in Ontario [16]. Given the presence of the same recombinant types in both the Chicago and Memphis cohorts, we propose that some of the mosaic viruses identified in this study might represent CRFs of FIV. However, we cannot exclude the possibility that the same recombinant forms could have arisen independently in distinct epidemiological areas on multiple occasions.

It is intriguing that clade A sequences were more likely to have a mosaic composition than those which were originally assigned to clade B. It has been proposed that clade B viruses are evolutionarily older, more host adapted and less pathogenic [20]. It is possible that the later emerging clade A viruses were able to superinfect cats and to recombine with already existing clade B viruses, resulting in the emergence of multiple Clade A/B recombinants in which the *env* gene predominantly comprised clade A derived sequences. These A/B recombinant strains hypothetically possessed equivalent or greater fitness compared to the parental A strains and were fixed in the population as CRFs of FIV. In contrast, A/B recombinants that were predominantly clade B derived were conspicuously rare, suggesting that these recombinants incurred a fitness cost.

Our analyses revealed the existence of at least one common recombination break point, located at the stem of the V1/V2 loop of the SU of FIV Env. Common recombination breakpoints, or hotspots, have been reported in both FIV [16] and HIV [5,6,9,31-33] infections. The identification of common recombination hotspots was limited since only *env* sequences were examined and it is likely that recombination processes within *env* were accompanied by recombination events elsewhere in the genome, such that additional, as yet unidentified, breakpoints exist [16,17].

Although recombination occurs frequently *in vitro* [34-36], it has been suggested that recombination breakpoints are driven by selection rather than being hotspots of RT template switching [4]. The vast majority of recombinant viruses display low fitness and do not survive within the host [37]. The recombinant *env* variants identified in our study must therefore have suffered no fitness cost, or might even have possessed some fitness advantage, compared to parental sequences.

The results of the present study highlight the risk of examining partial gene sequences. The detection of recombination increases with the nucleotide length screened and therefore the majority of previous studies (which examined only short 500-700 bp fragments of the *env* gene) underestimated the role of recombination using a simplified phylogenetic classification. Applying the common approach of examining the V3-V5 hypervariable regions of the *env* gene will likely result in the misidentification of intra- and inter-subtype recombinants and erroneous subtype assignment. For example, the recombinant structure of viruses such as M2, M33 or P7 would have been misclassified if only 700 bp fragments of the *env* had been examined. Indeed, previous studies focusing solely on the V3-V5 region of *env* [24,38] did not take into account the possibility of recombination which might have altered the classification of FIVs [17].

The recombination breakpoint separating the unusually long leader region from the remainder of the *env* gene is of particular interest. Several recombinant *env* sequences were identified in which the leader/signal region of *env* clustered with clade B while the other spans clustered with clade A. Given the location of this recombination breakpoint, together with the relatively high number of positively selected sites and the highest evolutionary rate of this span [28], the leader region may have a significant function in the viral life cycle and immune evasion.

Sequences isolated from the majority of animals formed highly monophyletic groups, suggesting that viruses were not transmitted between cats. Sequential sequences from three animals displayed incongruent phylogenetic assignment. Since the cats were housed together with other FIV infected cats, their shared accommodation may have led to infection with additional viruses, including transmission from unsampled cats, resulting in the potential turnover of the viral population. Given the relatively small number of sequences isolated following the postulated transmission event and sampling bias, it is possible that primary sequences remained but were less abundant than more recently acquired strains.

Potential limitations of this study include PCR sampling and cloning bias [39,40]. However, in contrast to previous studies, a high fidelity DNA polymerase (Phusion**®**) was used in place of the error prone *Taq* polymerase to avoid mutations arising during the PCR amplification of *env* sequences. Furthermore, in order to minimize template switching during the PCR, three independent amplifications were set up from each blood sample. The presence of similar recombinants, particularly those with shared recombination points, over a period of 18 months provides strong evidence that the amplicons identified were indeed circulating sequences rather than products of PCR-induced errors or polymerase template switching.

## Conclusions

Recombinant *env* sequences sharing a common breakpoint separating the leader/signal region from the remaining part of *env* were highly abundant in naturally infected cats in the USA. This finding is intriguing, particularly since the feline and ungulate lentiviruses possess unusually long leader/signal sequences compared to primate retroviruses [18]. The location of the identified recombination breakpoint suggests that the leader region of the FIV *env* could play an important role in virus biology and immune evasion, as has been described for signal sequences in other viruses [41]. These findings broaden our understanding of retroviral evolution and illustrate the significant role of recombination in generating viral diversity at the population level. Here we provide evidence for the existence of CRFs in two geographically distant American cities. The lack of information about CRFs of FIV in the field has wider implications than just the classification of FIV, since it poses significant questions about the likely efficacy of the current FIV vaccine. The degree of protection against recombinant viruses provided by commercial FIV vaccination is unknown. Furthermore, the existence of recombinant sequences has implications for the molecular diagnosis of FIV infection. CRFs of FIV, including some as yet unidentified, may remain undetected by PCR-based diagnostic tests [42,43] currently in use to distinguish FIV-vaccinated and FIV-infected cats in countries where the vaccine is widely available [44]. Further phylogenetic studies, ideally of two neighbouring genes or the whole genome of various strains of FIV from diverse geographic locations will be required to classify the virus more accurately, to optimize diagnostic protocols and to inform the development of an improved FIV vaccine.

## Methods

### FIV ORF *env* sequences

FIV were isolated from three serial blood samples collected at 6 monthly intervals from naturally infected domestic cats (n = 43). Cats were enrolled into the study based on a history of FIV diagnosis by ELISA (SNAP FIV/FeLV Combo Test, IDEXX), regardless of breed, age, sex or health status. All cats were FeLV antigen negative (SNAP FIV/FeLV Combo Test, IDEXX). FIV-positive status was confirmed by virus isolation [45]. Within the study group, 27 cats were homed together and cared for in a large multi-cat household in Memphis, TN, USA, where FIV-positive and FIV-negative cats were housed indoors with unrestricted access to one another. The remaining 16 cats were living in single households in Chicago, IL, USA with exception of five cats: two cats (P7 and P4) had been rehomed together and were living in the same household; one cat (P9) had been rehomed with another FIV-positive cat not enrolled in the study; and one cat (P13) had been rehomed with another FIV-negative cat; and one cat (P21) was housed with another two FIV-positive cats in the rehoming centre.

Cats from two cohorts displayed contrasting clinical outcomes of infection. The clinical signs were mild or unapparent in the Chicago cohort, while the outcome of infection in Memphis was dramatically different, with mortality rate reaching 63% and lymphoma being the most common cause of death [28].

The study and its aims were reviewed and approved by the University of Glasgow Ethics Committee and the Purdue Animal Care and Use Committee. Cat owners provided written informed consent for their participation in the study.

Multiple full length FIV *env* genes (~2500 bp) were amplified directly from whole blood using a nested PCR protocol [see Additional file 7: Table S4]. First round PCR products were amplified by Phusion® Blood Direct II Polymerase (Thermo Fisher Scientific), followed by direct nucleic acid sequence determination. The nucleic acid sequence of the first-round PCR product informed primer design for the second round PCR, which was performed using High Fidelity PCR Master (Roche). Strain-specific primers for second round PCR incorporated restriction sites for subsequent cloning into the eukaryotic expression vector VR1012 [46] and transformation into *E. coli* MAX Efficiency® DH5α™ Competent Cells (Invitrogen). Each VR1012-FIV *env* construct was sequenced using Big Dye Terminator v1.1 kit (Applied Biosystems) on an Applied Biosystems 3130xl capillary sequencer. Special measures were taken to avoid the possibility of contamination, both in the clinical and the laboratory settings: cats were double identified prior to blood sampling; PCR reactions were prepared in a designated UV treated room; and fresh, unopened reagents were used at each separate time point throughout the 18 month study period.

### Multiple sequence alignment

There were 355 serial *env* sequences from 43 cats available for analysis from the two cohorts [see Additional file 5: Table S2]. The number of sequences varied according to the availability of follow-up samples, because of the 63% mortality rate in the Memphis cohort during the study period [28]. Multiple sequence alignments were conducted using the Muscle algorithm [47] in MEGA5 [48]. Final alignments were curated manually to ensure re-positioning of incorrect gaps in sequences of variable lengths.

### Phylogenetic trees

Maximum likelihood (ML) trees were constructed in MEGA5 [48] under the HKY nucleotide substitution model, selected through jMODELTEST [49]. Statistical support for the ML trees was estimated using 1000 bootstrap replicates [50].

### Recombination testing

Sequences from the study group (n = 355), together with reference full-length *env* sequences obtained from GenBank (n = 19), were subjected to rigorous five-fold recombination testing: 1) The initial recombination analysis included the entire data set and was performed with the Datamonkey webserver [51], employing Single Breakpoint (SBP) and Genetic Algorithm Recombination Detection (GARD) methods [52] and using the Pairwise Homoplasy Index in SPLITSTREE [53]; 2) The mosaic structure of recombinant sequences was confirmed by subsequent GARD analysis focussing only on study group sequences identified previously as recombinants, to achieve higher resolution (maximum likelihood trees for each recombination span identified by GARD and assessed by Akaike Information Criterion (AIC) [54] were constructed on the Datamonkey webserver); 3) The mosaic structure of recombinant sequences was tested by employing probabilistic approach implemented in jpHMM [55]; 4) Major and minor parents for mosaic recombinant sequences were identified by the RDP detection method [56] and confirmed for the complete dataset by SPLITSTREE network [53]; and 5) Representative figures visualizing recombination breakpoints were generated in SimPlot v 3.5.1 [57].

## Additional files

Additional file 1:
**The Maximum Likelihood (ML) tree constructed under HKY substitution model using the entire data set.** The tree is saved in the NEWICK format and can be opened in FigTree v 1.3.1 (http://tree.bio.ed.ac.uk/). Additional file 2: Figure S1. ML trees representing phylogenetic inference of seven GARD determined spans. Additional file 3: Table S1. Comparison of GARD and jpHMM identified recombination breakpoints. Additional file 4: Figure S2. Similarity analysis of: A) Memphis A/B recombinant sequence (M31) and B) Chicago A/B recombinant sequence (P21C). Additional file 5: Table S2. Number of sequences isolated from each time point from the US cats. Additional file 6: Table S3. FIV load, (genomes/mL blood). Additional file 7: Table S4. Primers.

## Abbreviations

FIV: Feline immunodeficiency virus
RT: Reverse transcriptase
URF: Unique recombinant form
CRF: Commonly circulating recombinant form
GARD: Genetic algorithm recombination detection
AIC: Akaike information criterion
SBP: Single breakpoint
ML: Maximum likelihood
bp: Base pair
