# Supplementary material for: Feline immunodeficiency virus (FIV) env recombinants are common in natural infections
Source: Retrovirology. 2014 Sep 17;11:80. doi: 10.1186/s12977-014-0080-1 (PMC4180853; doi:10.1186/s12977-014-0080-1)

**Additional file 2 – Figure S1** ML trees for each of seven GARD determined spans. Trees A, B, C, D, E, F and G represent phylogenetic inference of segments 1) 1-354; 2) 355-564; 3) 565-1272; 4) 1273-1608; 5) 1609-1868; 6) 1869-2255 and 7) 2256-2604 respectively. Note various clade assignments for each sequence segment from the same cat in each of the trees (e.g. P21C). The tips on all seven trees are coloured according to the phylogenetic assignment of span 3. Bootstrap supports above 75 are shown.

1. Span 1


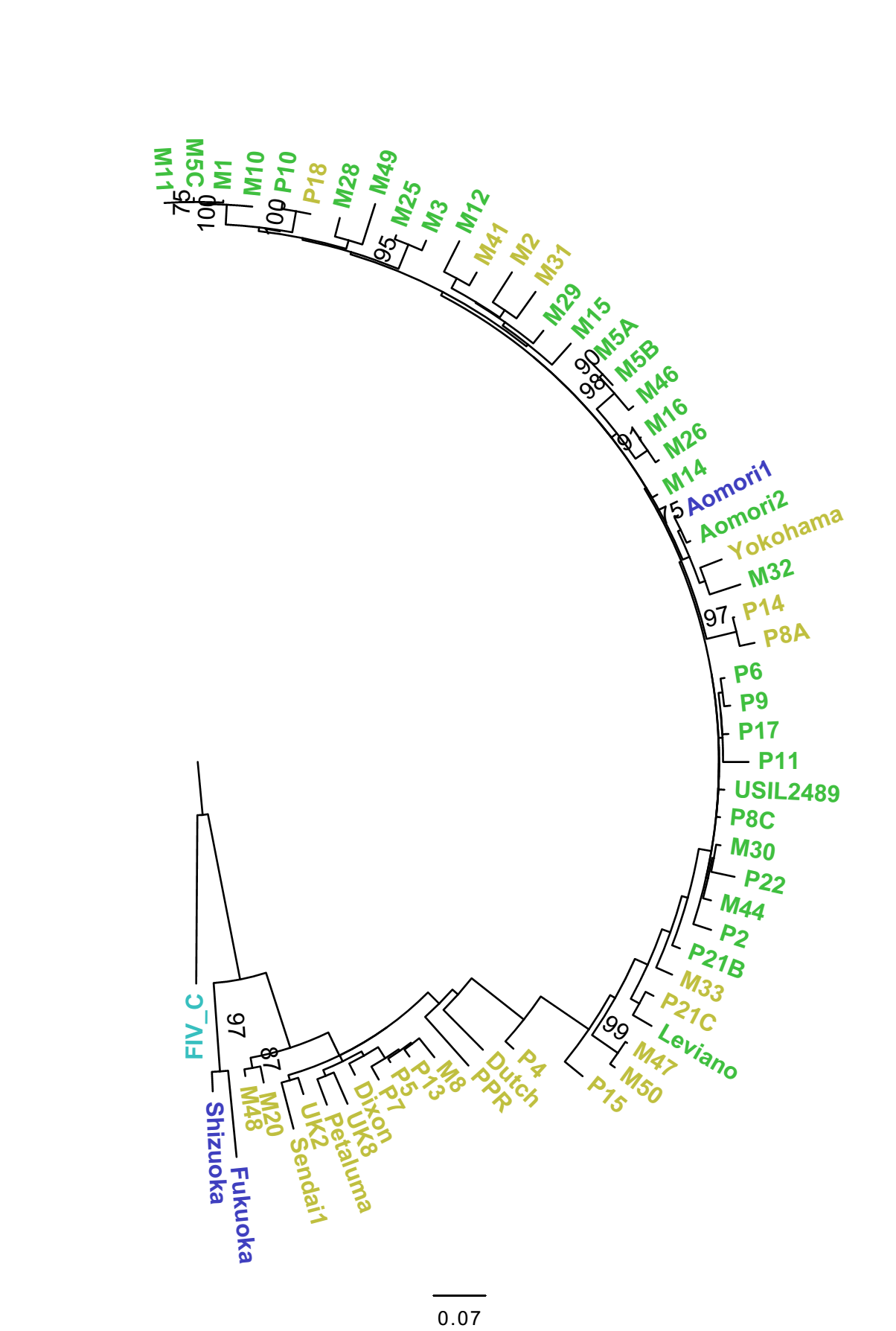


1. Span 2


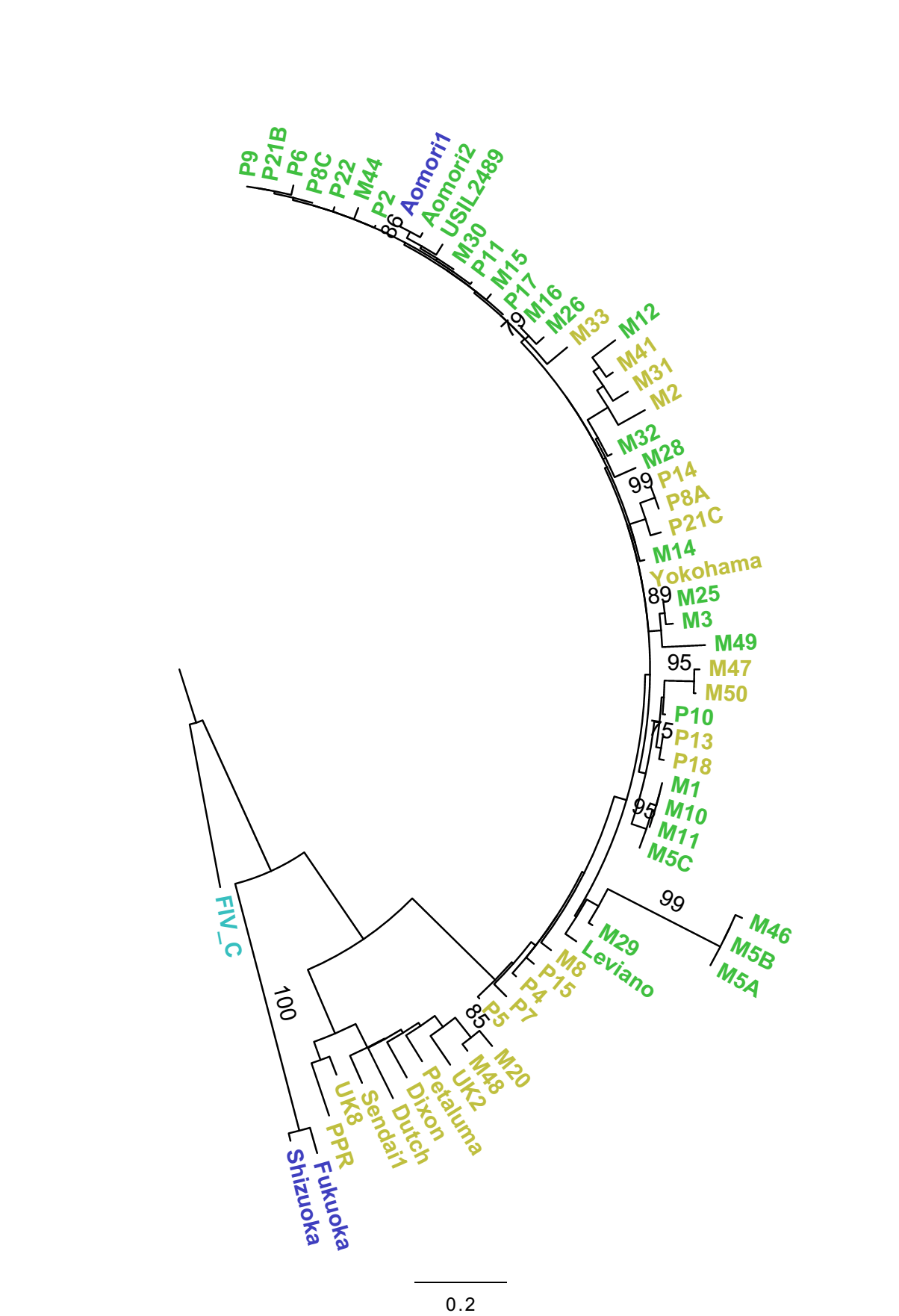


1. Span 3


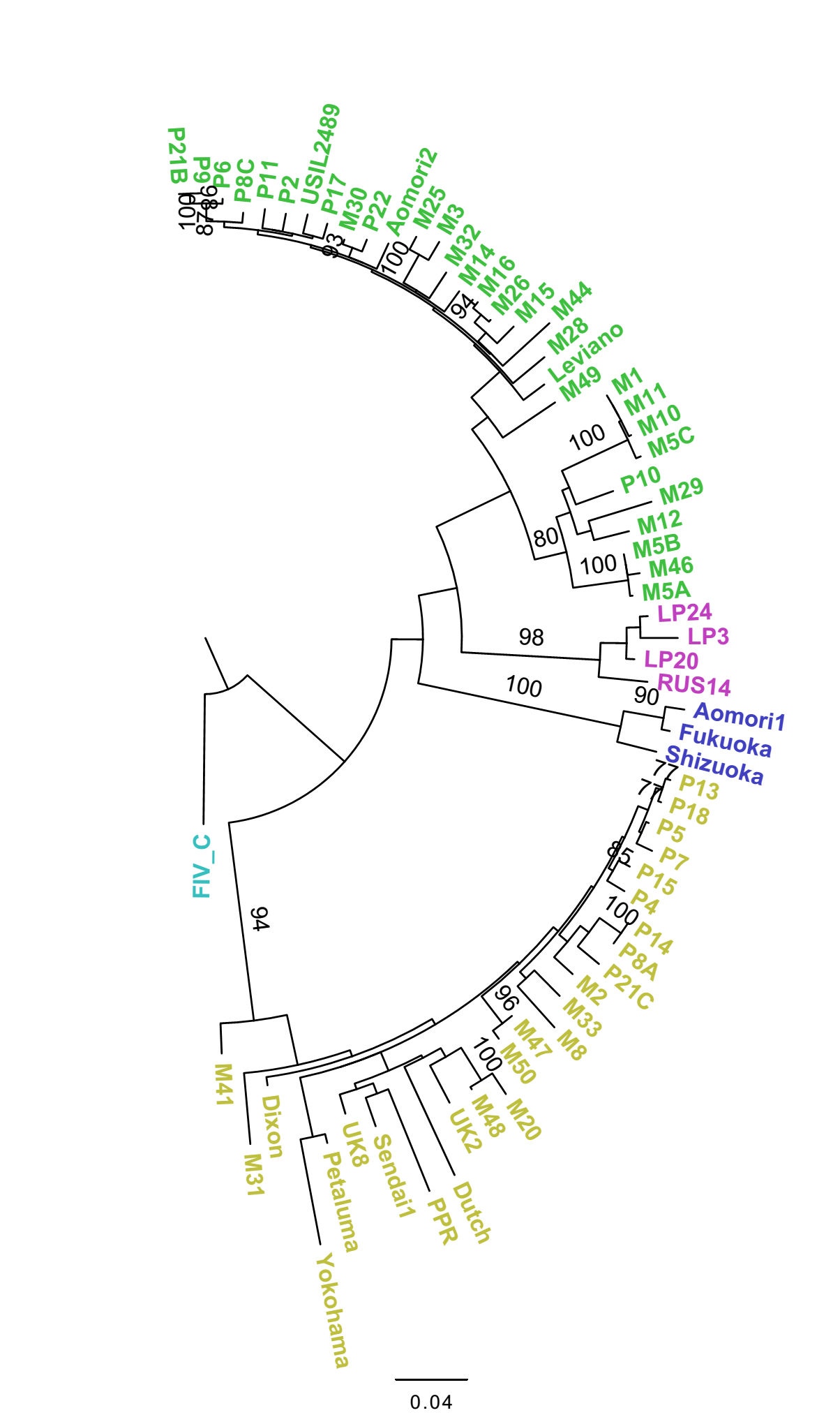


1. Span 4


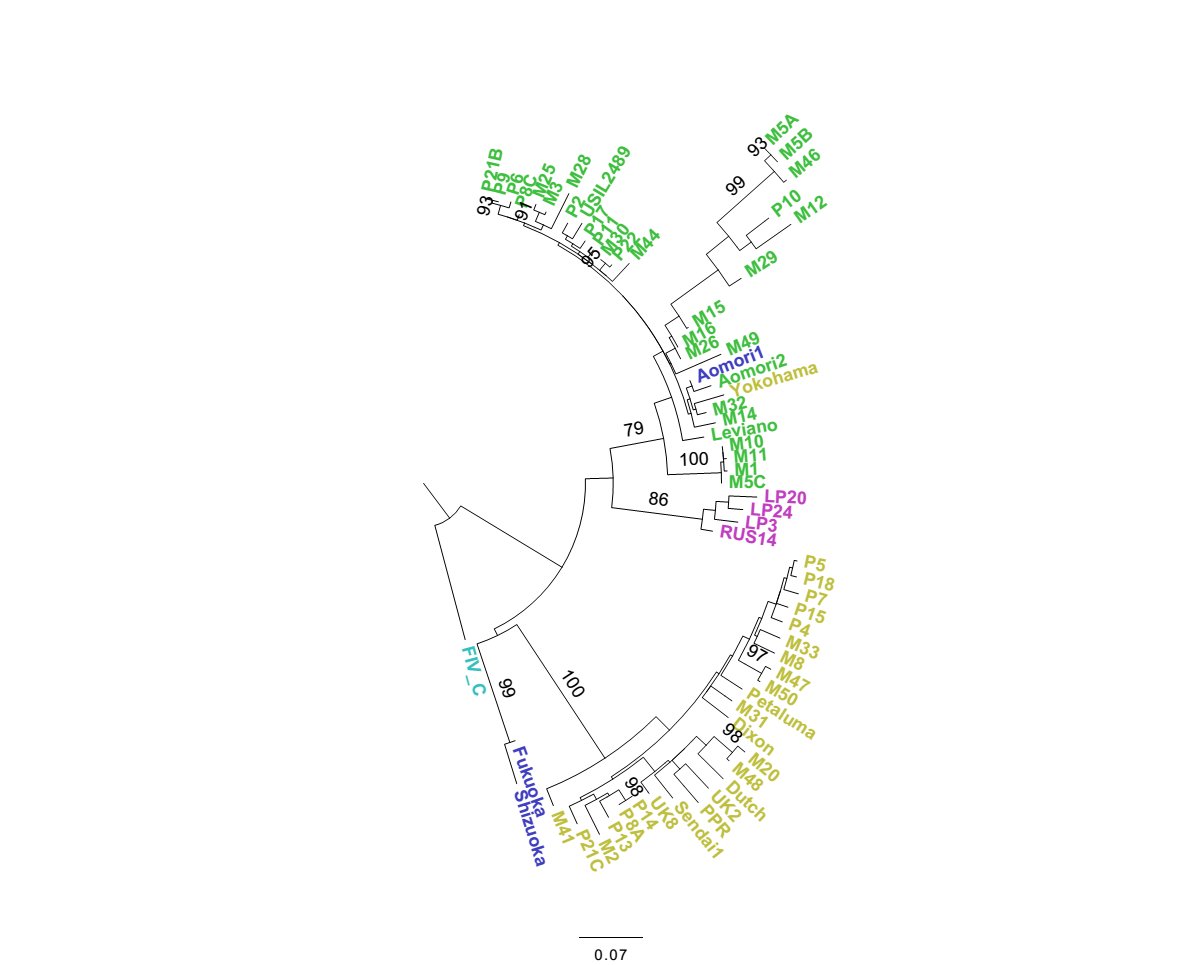


1. Span 5


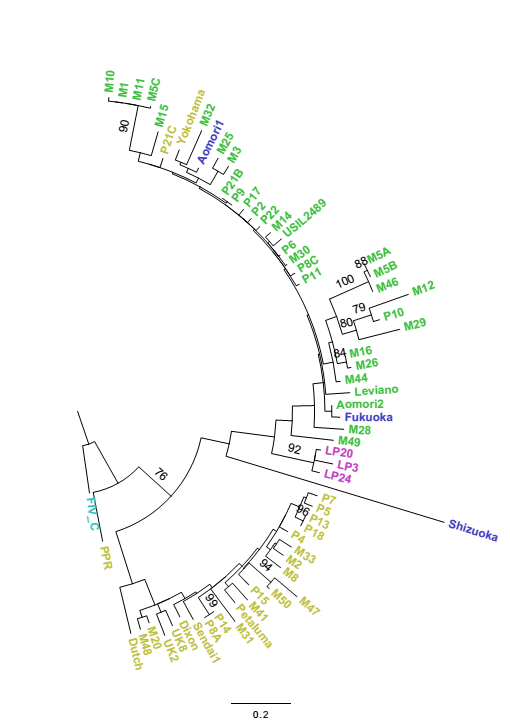


1. Span 6


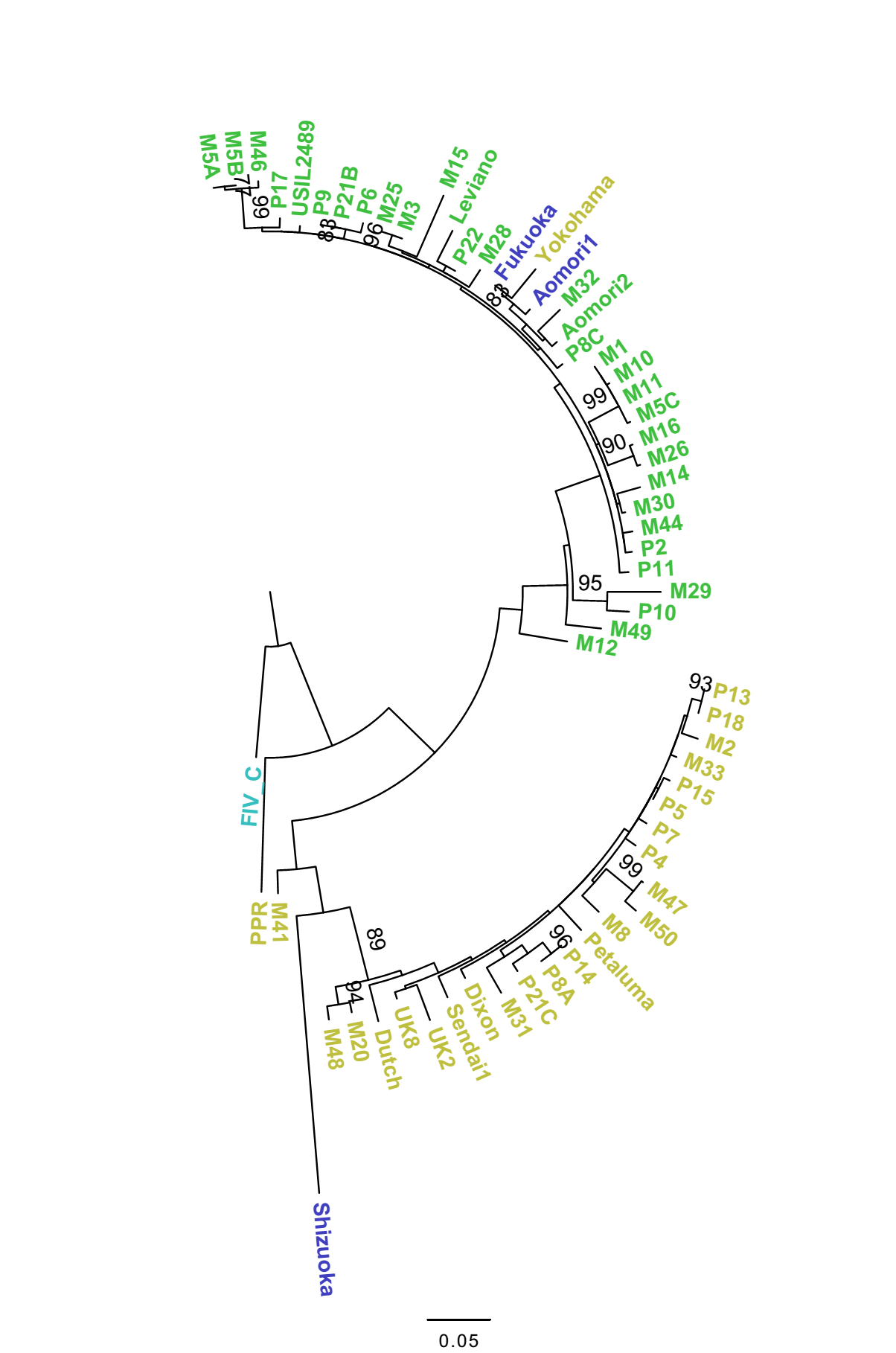


1. Span 7


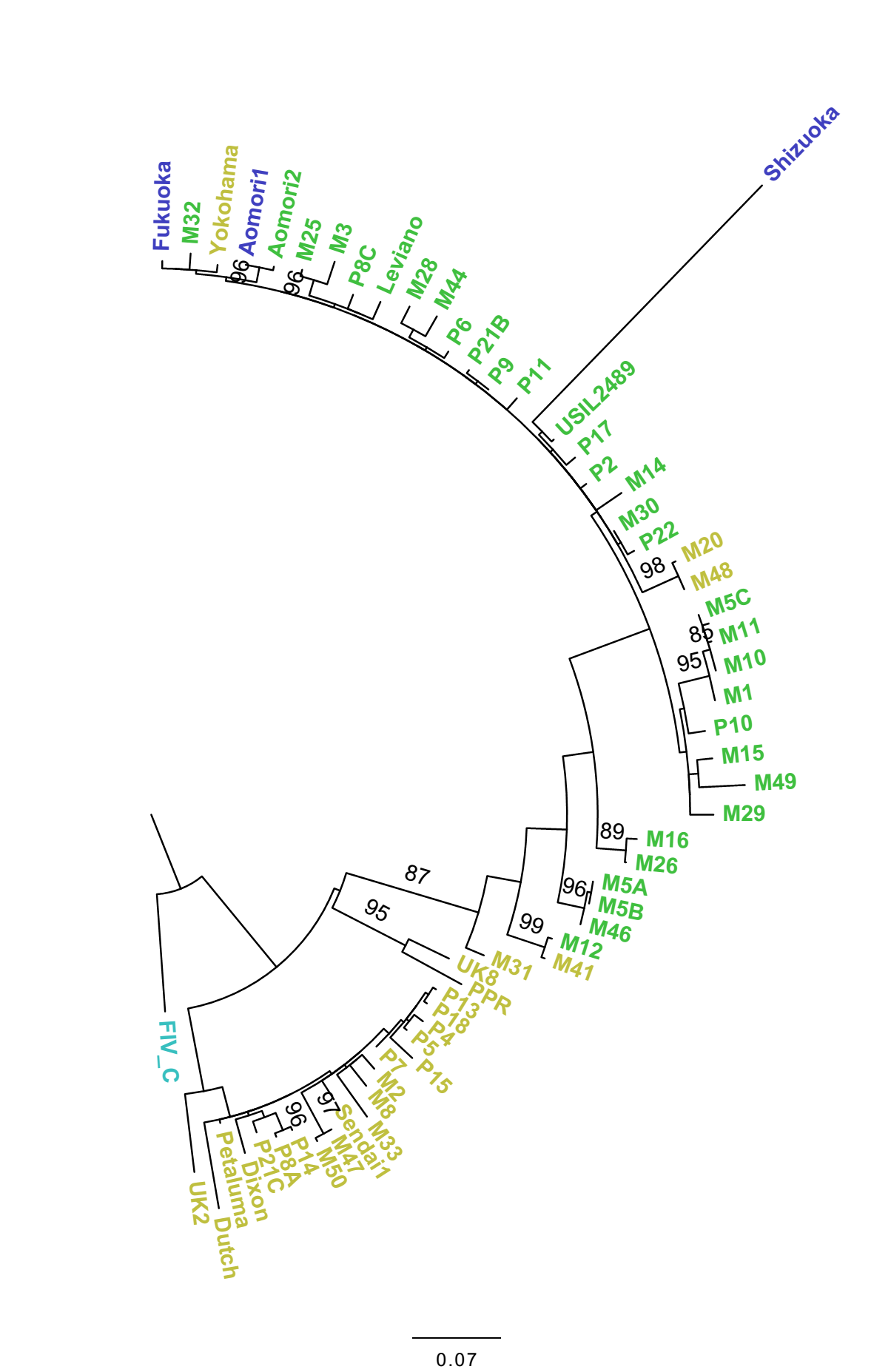

Supplement: Additional file 2: Figure S1. — ML trees representing phylogenetic inference of seven GARD determined spans. [file 12977_2014_80_MOESM2_ESM.docx]
