# Supplementary material for: Feline immunodeficiency virus (FIV) env recombinants are common in natural infections
Source: Retrovirology. 2014 Sep 17;11:80. doi: 10.1186/s12977-014-0080-1 (PMC4180853; doi:10.1186/s12977-014-0080-1)
Supplement: Additional file 3: Table S1. — Comparison of GARD and jpHMM identified recombination breakpoints. [file 12977_2014_80_MOESM3_ESM.docx]

**Additional file 3 - Table S1** Comparison of GARD and jpHMM identified recombination breakpoints. Additional breakpoints identified by jpHMM but not by GARD are highlighted in red.

| Cat | **GARD results** | | | **jpHMM results** | | | |
| --- | --- | --- | --- | --- | --- | --- | --- |
|  | Form | No of breakpoints | Breakpoint location | Form | No of breakpoints | Breakpoint location | Breakpoint interval |
| M1 | B |  |  | B |  |  |  |
| M2 | B-A | 1 | 564 | B-A | 1 | 599 | 577-605 |
| M3 | B |  |  | B |  |  |  |
| M5A | B |  |  | B |  |  |  |
| M5B | B |  |  | B |  |  |  |
| M5C | B |  |  | B |  |  |  |
| M8 | A-B-A | 2 | 354; 564 | A-B-A | 2 | 380; 560 | 367-392; 553-560 |
| M10 | B |  |  | B |  |  |  |
| M11 | B |  |  | B |  |  |  |
| M12 | B |  |  | B |  |  |  |
| M14 | B |  |  | B |  |  |  |
| M15 | B |  |  | B |  |  |  |
| M16 | B |  |  | B |  |  |  |
| M20 | A-B | 1 | 2256 | B-A-B | 2 | 27; 2226 | 24-27; 2198-2249 |
| M25 | B |  |  | B |  |  |  |
| M26 | B |  |  | B |  |  |  |
| M28 | B |  |  | B |  |  |  |
| M29 | B |  |  | B |  |  |  |
| M30 | B |  |  | B |  |  |  |
| M31 | B-A-B | 2 | 564; 2255 | B-A-B-A-B | 4 | 554; 933; 1043; 2247 | 547-563; 931-953; 1040-1073; 2248-2278 |
| M32 | B |  |  | B |  |  |  |
| M33 | B-A | 1 | 564 | A-B-A | 2 | 51; 560 | 30-64; 553-560 |
| M41 | B-A-D-B | 3 | 564, 1869, 2256 | B-A-B-A-B | 4 | 563; 1824; 1995; 2205 | 556-563; 1823-1837; 1994-2039; 2206-2273 |
| M44 | B |  |  | B |  |  |  |
| M46 | B |  |  | B |  |  |  |
| M47 | B-A | 1 | 564 | A-B-A | 2 | 103; 560 | 104-128; 553-560 |
| M48 | A-B | 1 | 2256 | A-B |  | 2220 | 2221-2243 |
| M49 | B |  |  | B |  |  |  |
| M50 | B-A | 1 | 564 | A-B-A | 2 | 103; 560 | 104-128; 553-560 |
| P2 | B |  |  | B |  |  |  |
| P4 | A-B-A | 2 | 354; 564 | A-B-A | 2 | 214; 560 | 212-224;553-560 |
| P5 | A-B-A | 2 | 354, 564 | A-B-A | 2 | 380; 560 | 367-391; 553-560 |
| P6 | B |  |  | B |  |  |  |
| P7 | A-B-A | 2 | 354; 564 | A-B-A | 2 | 380; 560 | 367-391; 544-560 |
| P8A | B-A | 1 | 564 | B-A | 1 | 599 | 577-616 |
| P8C | B |  |  | B |  |  |  |
| P9 | B |  |  | B |  |  |  |
| P10 | B |  |  | B |  |  |  |
| P11 | B |  |  | B |  |  |  |
| P13 | A-B-A | 2 | 354; 564 | A-B-A | 2 | 380; 560 | 367-391; 553-560 |
| P14 | B-A | 1 | 564 | B-A | 1 | 602 | 580-619 |
| P15 | B-A | 1 | 564 | A-B-A | 2 | 103; 554 | 104-134; 550-560 |
| P17 | B |  |  | B |  |  |  |
| P18 | B-A | 1 | 564 | B-A | 1 | 563 | 556-563 |
| P21B | B |  |  | A-B | 1 | 21 | 22-24 |
| P21C | B-A-B-A | 3 | 564, 1609, 1869 | A-B-A-B-A | 4 | 60; 599; 1548; 1839 | 60-77; 577-616; 1543-1576; 1828-1839 |
| P22 | B |  |  | B |  |  |  |
