# Supplementary material for: Feline immunodeficiency virus (FIV) env recombinants are common in natural infections
Source: Retrovirology. 2014 Sep 17;11:80. doi: 10.1186/s12977-014-0080-1 (PMC4180853; doi:10.1186/s12977-014-0080-1)
Supplement: Additional file 4: Figure S2. — Similarity analysis of: A) Memphis A/B recombinant sequence (M31) and B) Chicago A/B recombinant sequence (P21C). [file 12977_2014_80_MOESM4_ESM.docx]

| A | B |
| --- | --- |
| 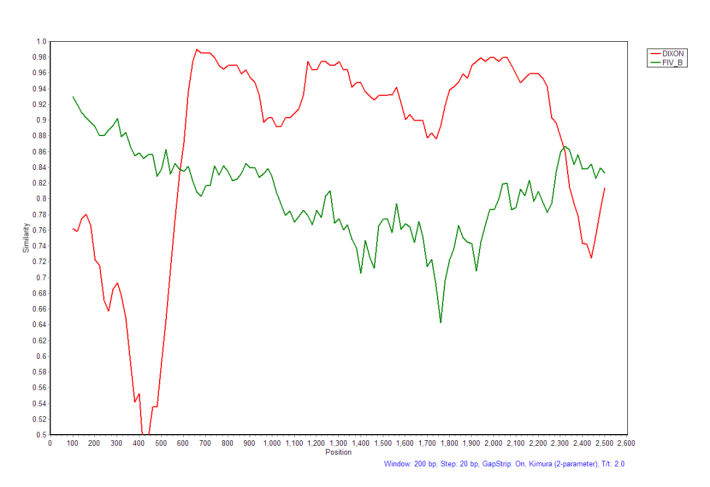 | 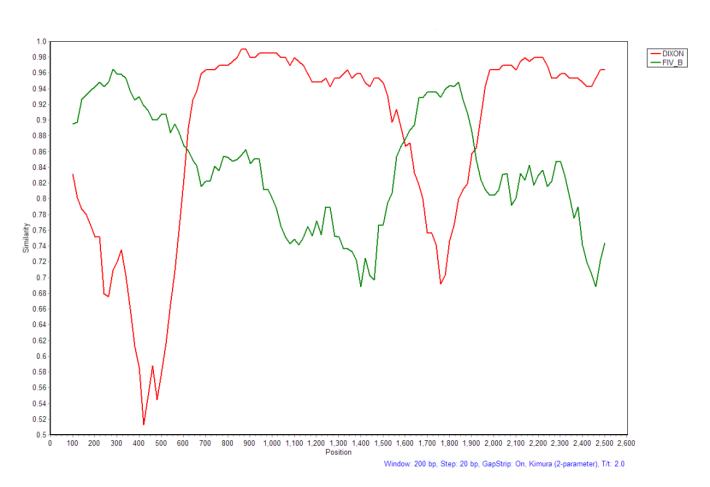 |

## Additional file 4 - Figure S2 - Similarity analysis of: A) Memphis A/B recombinant sequence (M31) and B) Chicago A/B recombinant sequence (P21C) Each line represents the percentage similarity of the query sequence to the reference sequences (clade A, DIXON [GenBank:L00608.1] in red and clade B, FIV_B [GenBank:U11820.1] in green). Recombination breakpoints are placed at the intersections of the red and green curves on the X axis (positions 562 and 2287 for M31; and positions 576, 1576 and 1947 for P21C). The window size in SimPlot was set for 200 bp.
