# Supplementary material for: Feline immunodeficiency virus (FIV) env recombinants are common in natural infections
Source: Retrovirology. 2014 Sep 17;11:80. doi: 10.1186/s12977-014-0080-1 (PMC4180853; doi:10.1186/s12977-014-0080-1)
Supplement: Additional file 5: Table S2. — Number of sequences isolated from each time point from the US cats. [file 12977_2014_80_MOESM5_ESM.docx]

**Additional file 5 - Table S2** Number of sequences isolated from each time point from the US cats. There were 228 *envs* available from 27 cats in the Memphis (M) cohort and 127 *env* gene sequences available for analysis from 16 Chicago (P) cats. * including sequences with premature stop codons.

| **No** | **Cat** | **Time point** | | | **Available**  **sequences** | **No** | **Cat** | **Time point** | | | **Available**  **sequences** |
| --- | --- | --- | --- | --- | --- | --- | --- | --- | --- | --- | --- |
|  |  | A | B | C |  |  |  | A | B | C |  |
| **1** | M1 | 2(*3) | 0 | 3 | 5(*6) | **23** | M46 | 3 | 3 | 3 | 9 |
| **2** | M2 | 0 | 0 | 2(*3) | 2(*3) | **24** | M47 | 3 | 2(*3) | 2(*3) | 7(*9) |
| **3** | M3 | 2(*3) | 0 | 0 | 2(*3) | **25** | M48 | 0(*3) | 0 | 0 | 0(*3) |
| **4** | M5 | 4 | 3 | 3 | 10 | **26** | M49 | 3 | 0 | 3 | 6 |
| **5** | M8 | 0 | 0 | 2 | 2 | **27** | M50 | 4(*5) | 0 | 0 | 4(*5) |
| **6** | M10 | 2 | 0 | 0 | 2 | **28** | P2 | 3 | 15 | 0 | 18 |
| **7** | M11 | 3 | 3 (*6) | 18 | 24 (*27) | **29** | P4 | 3 | 4 | 3(*4) | 10(*11) |
| **8** | M12 | 0(*1) | 0 | 0(*2) | 0(*3) | **30** | P5 | 2(*3) | 3(*4) | 3 | 8(*10) |
| **9** | M14 | 3 | 1(*3) | 5 | 9(*11) | **31** | P6 | 3 | 2 | 5 | 10 |
| **10** | M15 | 3 | 2(*3) | 3 | 8(*9) | **32** | P7 | 0(*3) | 1 | 1(*4) | 2(*8) |
| **11** | M16 | 4 | 0 | 2 | 6 | **33** | P8 | 5 | 0 | 0(*3) | 5(*8) |
| **12** | M20 | 1(*2) | 0 | 0 | 1(*2) | **34** | P9 | 3 | 0 | 3 | 6 |
| **13** | M25 | 3 | 0 | 3 | 6 | **35** | P10 | 3 | 1 | 0 | 4 |
| **14** | M26 | 3 | 0 | 3 | 6 | **36** | P11 | 0 | 2 | 4 | 6 |
| **15** | M28 | 3 | 5 | 3 | 11 | **37** | P13 | 3 | 3 | 3 | 9 |
| **16** | M29 | 0 | 0 | 4 | 4 | **38** | P14 | 2 | 4(*5) | 3 | 9(*10) |
| **17** | M30 | 5 | 15(*16) | 5 | 25(*26) | **39** | P15 | 0(*2) | 0(*3) | 0(*3) | 0(*8) |
| **18** | M31 | 1(*2) | 0(*2) | 13 | 14(*17) | **40** | P17 | 2 | 4 | 2 | 8 |
| **19** | M32 | 3 | 0 | 0 | 3 | **41** | P18 | 0 | 1 | 0 | 1 |
| **20** | M33 | 7(*8) | 14(*16) | 0 | 21(*24) | **42** | P21 | 0 | 3 | 2(*3) | 5(*6) |
| **21** | M41 | 3 | 0 | 10 | 13 | **43** | P22 | 0 | 0 | 3 | 3 |
| **22** | M44 | 2 | 0 | 0 | 2 |  |  |  |  | Total | 306 (*355) |
