# Supplementary material for: Feline immunodeficiency virus (FIV) env recombinants are common in natural infections
Source: Retrovirology. 2014 Sep 17;11:80. doi: 10.1186/s12977-014-0080-1 (PMC4180853; doi:10.1186/s12977-014-0080-1)
Supplement: Additional file 6: Table S3. — FIV load, (genomes/mL blood). [file 12977_2014_80_MOESM6_ESM.docx]

**Additional file 6 –** **Table S3** FIV load, (genomes/mL blood). FIV load was measured for each time point (A, B and C) using a commercially available PCR test (IDEXX FIV RealPCR Test, IDEXX Laboratories, West Sacramento, CA). The assay detects the presence of viral nucleic acid, including both genomic DNA and viral RNA, in peripheral blood leukocytes with 80.5% sensitivity and 99.9% specificity (IDEXX Laboratories, West Sacramento, CA). n/a= not available.

| **No** | **Cat** | **Viral load** | | | **No** | **Cat** | **Viral load** | | |
| --- | --- | --- | --- | --- | --- | --- | --- | --- | --- |
|  |  | **A** | **B** | **C** |  |  | **A** | **B** | **C** |
| **1** | M1 | n/a | n/a | n/a | **23** | M46 | 418671 | n/a | n/a |
| **2** | M2 | 56867 | n/a | n/a | **24** | M47 | 140562 | n/a | n/a |
| **3** | M3 | 13991 | n/a | n/a | **25** | M48 | 3792122 | n/a | n/a |
| **4** | M5 | 19225 | n/a | n/a | **26** | M49 | 1383178 | n/a | n/a |
| **5** | M8 | 24147 | n/a | n/a | **27** | M50 | 23489 | n/a | n/a |
| **6** | M10 | 7724 | n/a | n/a | **28** | P2 | n/a | n/a | n/a |
| **7** | M11 | 1666782 | n/a | n/a | **29** | P4 | 673 | 179 | NEGATIVE |
| **8** | M12 | 418671 | n/a | n/a | **30** | P5 | 2911 | 3076 | 73801 |
| **9** | M14 | 418671 | n/a | n/a | **31** | P6 | 741 | 164 | 1596164 |
| **10** | M15 | 43138 | n/a | n/a | **32** | P7 | 1099 | 421 | 39633 |
| **11** | M16 | 13423 | n/a | n/a | **33** | P8 | 395 | 111 | 1061881 |
| **12** | M20 | 31395 | n/a | n/a | **34** | P9 | 18796 | 80 | 144 |
| **13** | M25 | 64843 | n/a | n/a | **35** | P10 | 540 | 43 | 7710 |
| **14** | M26 | 176551 | n/a | n/a | **36** | P11 | 18796 | 97 | 1236165 |
| **15** | M28 | 24483 | n/a | n/a | **37** | P13 | 2104 | 229 | 8261 |
| **16** | M29 | 2540 | n/a | n/a | **38** | P14 | 692 | 366 | 297900 |
| **17** | M30 | 2703217 | n/a | n/a | **39** | P15 | 767 | NEGATIVE | 240 |
| **18** | M31 | 41387 | n/a | n/a | **40** | P17 | 668 | n/a | 1664 |
| **19** | M32 | 16176 | n/a | n/a | **41** | P18 | n/a | n/a | 16946 |
| **20** | M33 | 2629546 | n/a | n/a | **42** | P21 | n/a | 45 | 1077 |
| **21** | M41 | 14583 | n/a | n/a | **43** | P22 | n/a | 43 | NEGATIVE |
| **22** | M44 | 179007 | n/a | n/a |  |  |  |  |  |
